# Discontinuation of metformin to prevent metformin-induced high colonic FDG uptake: is 48 h sufficient?

**DOI:** 10.1007/s12149-020-01509-z

**Published:** 2020-08-19

**Authors:** Nanno Schreuder, Hedwig Klarenbeek, Brian N. Vendel, Pieter L. Jager, Jos G. W. Kosterink, Eugène P. van Puijenbroek

**Affiliations:** 1 Groningen Research Institute of Pharmacy, Pharmacotherapy, Epidemiology and Economics, University of Groningen, Antonius Deusinglaan 1, Groningen, The Netherlands; 2 GE Healthcare Radiopharmacy Zwolle, Zwolle, The Netherlands; 3 Department of Nuclear Medicine, Isala Hospital, Zwolle, The Netherlands; 4 Department of Clinical Pharmacy and Pharmacology, University of Groningen, University Medical Center Groningen, Groningen, The Netherlands; 5 Netherlands Pharmacovigilance Centre Lareb, ’s Hertogenbosch, The Netherlands

**Keywords:** [^18^F]fluorodeoxyglucose, PET/CT, Metformin, Radiopharmaceuticals, Drug interaction

## Abstract

**Objective:**

In this retrospective, single-center observational study, we investigated whether discontinuing metformin for at least 48 h prevents metformin-induced [^18^F]fluorodeoxyglucose (FDG) uptake in all segments of the colon.

**Methods:**

Patients with type 2 diabetes who were using metformin before undergoing an FDG PET/CT scan were included. Two groups were created: patients who discontinued metformin for less than 48 h (< 48 h group) and patients who discontinued metformin for between 48 and 72 h (≥ 48 h group). A control group comprised non-diabetic patients who were not using metformin before undergoing an FDG PET/CT. We visually scored the uptake of FDG in four segments of the colon—the ascendens, transversum, descendens, and rectosigmoid—using a four-point scale (1–4) and considered scores of 3 or 4 to be clinically significant.

**Results:**

Colonic FDG uptake in the ≥ 48 h group (*n* = 23) was higher than uptake in the control group (*n* = 96) in the colon descendens [odds ratio (OR) 14.0; 95% confidence interval (CI) 4.8–40.9; *p* value: 0.001] and rectosigmoid (OR 11.3; 95% CI 4.0–31.9; *p* value: 0.001), and there was no difference in the colon ascendens and transversum. Colonic FDG uptake in the < 48 h group (*n* = 25) was higher than uptake in the ≥ 48 h group (*n* = 23) in the colon transversum (OR 4.8; 95% CI 1.3–18.5; *p* value: 0.022) and rectosigmoid (*p* value: 0.023), and there was no difference in the colon ascendens and descendens.

**Conclusions:**

Discontinuing metformin for 48 h before undergoing an FDG PET/CT still gives a high uptake in the distal parts of the colon when compared with non-diabetic patients who are not using metformin. Discontinuing metformin for 48 h seems to be useful for scanning the more proximal segments of the colon.

## Introduction

[^18^F]fluorodeoxyglucose (FDG) positron emission tomography/computed tomography (PET/CT) is used in oncology for diagnosis, staging, restaging, and the assessment of response to therapy [1]. Several studies have found an increased FDG uptake—predominantly in the colon—in patients who are using metformin, an oral antidiabetic used in patients with diabetes mellitus type 2 [2–4]. Although FDG PET is not generally performed to study primary colorectal cancer, the increased uptake of FDG in the colon could still obscure lesions and significant findings may be missed. As it is estimated that the number of patients with diabetes mellitus will increase with an averaged annual growth of 2.7% [5], nuclear medicine physicians will see more patients who are on metformin.

The guidelines of the European Association of Nuclear Medicine (EANM) [6] recommend that patients continue to take antidiabetics around PET scanning. An exception is when PET scanning is combined with an intravenous contrast agent, in which case, metformin should be discontinued at the time of the procedure and 48 h thereafter to prevent metformin-associated lactic acidosis. The Society of Nuclear Medicine and Molecular Imaging (SNMMI) does not specify guidelines for PET procedures made in patients on oral antidiabetics [7]. However, several studies have suggested that metformin should be discontinued prior to the FDG PET scan [2–4], and have recommended feasible and optimal discontinuation periods of metformin [4, 8, 9] to limit the amount of FDG uptake. Even so, the results in these studies are contradictory. Studies by Oh et al. and Hamidizadeh et al. found that discontinuing metformin for 48 h reduced the intestinal FDG uptake in comparison to the group that continued metformin [4, 8]. Oh et al. concluded that discontinuing metformin 48 h before an FDG PET scan is an effective preparation [4]. In contrast, a study performed by Lee et al. demonstrated that FDG uptake in the distal colon remained high even after metformin had been discontinued for over 48 h [9].

As there is currently no consensus on the time at which metformin should be discontinued, we aimed to determine whether a metformin discontinuing period of ≥ 48 h is sufficient to prevent FDG uptake in all segments of the colon when compared with a control group of non-diabetic patients who are not using metformin. In other words, does discontinuing metformin in patients make their colonic FDG uptake the same as can be expected in patients who are not taking this drug? In addition, we assessed whether colonic FDG uptake differs between patients who have discontinued taking metformin < 48 h before the procedure versus those who have discontinued ≥ 48 h before.

## Methods

### Study design and patients

We conducted a retrospective, single-center observational study between January 2018 and September 2018 in diabetic patients who had been using metformin before undergoing an FDG PET/CT scan in Isala hospital, Zwolle, a tertiary referral and a 1103-bed regional hospital in The Netherlands. We compared these data with a control group of non-diabetic patients who had not been using metformin. As metformin is routinely discontinued in patients for 48 h, we received an ethical exemption in writing from the Medical Ethics Committee of the Isala hospital, in Zwolle in The Netherlands (Reference number 190701), as this study did not require formal approval according to Dutch law. However, all patients gave their approval for the use of their data for this evaluation, in agreement with Dutch privacy laws.

Diabetic patients who had been using metformin before undergoing an FDG PET/CT scan and gave approval for using their data for this study were included. We excluded patients with a known colonic malignancy or inflammatory bowel diseases in the medical records, patients who received PET scans that did not cover the abdomen, patients who had discontinued metformin for > 72 h, and cases with incomplete data, such as an unknown metformin discontinuation period or unknown metformin dosage. In patients who had received multiple scans within this period, only the first scan was analyzed. At the Isala hospital, Zwolle, patients who are using metformin are instructed to withhold metformin for 48 h prior to a PET/CT scan as usual care, but in practice, discontinuation periods may vary. Based on the actual reported time of discontinuing metformin, we created two groups: patients who had discontinued metformin for < 48 h (< 48 h group) and patients who had discontinued metformin for ≥ 48 to ≤ 72 h (≥ 48 h group). Patients in the control group were selected from non-diabetic patients who had not been using metformin and underwent an FDG PET/CT on the same or next day as the metformin patients. For each patient who had been using metformin, we included two consecutive patients in the control group.

For each patient, we recorded the following characteristics: age, sex, body mass index (BMI; kg/m^2^), blood glucose level (mmol/L), and FDG dose (MBq/kg). In the metformin group, we also asked patients about their daily metformin dose, insulin usage, and their self-reported discontinuation period of metformin (in hours).

### Patient preparation

All patients were instructed to fast for at least 6 h and—if applicable—to withhold insulin prior to the FDG injection, according to standard protocol. Immediately before the FDG injection, patients’ blood glucose levels were measured in capillary blood obtained from a finger-stick with an Accu-Chek glucometer (Roche diagnostics). When the patients’ blood glucose reached a level that was higher than 15 mmol/L, the PET/CT scan was canceled, and the patients were scheduled for another appointment with additional instructions or by emphasizing their adherence to the instructions, depending on the cause of the high blood glucose level prior to PET scanning.

### PET/CT acquisition and reconstitution

PET/CT scans were acquired on a Vereos PET/CT (Philips Healthcare) or Ingenuity TF PET/CT (Philips Healthcare). The injected FDG activity (*A*, MBq) was based on the body weight of the patients (*w*, kg) and the acquisition time per bed position (*t* in seconds) using the following quadratic equation: $A=5.2\frac{{w}^{2}}{t}$ (MBq). Patients were scanned 60 min after FDG injection. Images were acquired over 10–16-bed positions—taking 72/144 s each—based on a scan from the crown to mid-thigh or the crown to the middle of the lower leg. PET data were reconstructed in a 144 × 144 matrix size with a voxel size of 4 × 4 × 4 mm^3^ (representing a default transaxial field of view of 576 mm), using a default 3D ordered-subset iterative time-of-flight (TOF) reconstruction technique (Vereos PET/CT reconstitution settings: 3 iterations, 15 subsets, and a 3 mm Gaussian post-smoothing filter; Ingenuity TF PET/CT reconstitution settings: 3 iterations, 43 subsets, and a relaxation parameter of 1.0), fulfilling EANM Research Ltd (EARL) accreditation specifications [10]. A non-contrast CT scan that was used for attenuation correction was obtained using the following parameters: 120 kV, 40–200 mAs rotation time of 0.5 s, and 4 mm slice thickness.

### Data analysis

We randomized and anonymized the obtained PET scans through the use of specific settings of our picture archiving and communication (PACS) system (Sectra IDS7). This ensured that the researchers were blinded to the underlying study group conditions, such as the use of metformin. We visually analyzed the FDG uptake in the colon on dedicated workstations (Sectra IDS7). The following segments of the colon were investigated: the ascendens (from the cecum to the hepatic flexure), transversum (from the hepatic flexure to the splenic flexure), descendens (from the splenic flexure to the sigmoid colon), and rectosigmoid colon (from the sigmoid colon to the anus, excluding the rectal sphincter). The rectosigmoid colon was included, because the presence of rectal cancer and, thus, uptake in the rectum are not unusual [11]. The uptake in the rectal sphincter was excluded.

Two expert nuclear medicine physicians (B. V., P. J.) and one trained researcher (H.K.), who were blinded to the study group conditions, independently conducted the visual analysis. The four-point scale method of Gontier et al. [2] was used to score the uptake in the colon, whereby a score of 1 = lower uptake than background hepatic activity, a score of 2 = similar uptake to hepatic activity, 3 = moderately higher uptake than hepatic activity, and 4 = intense and diffuse uptake. A score of 3 or 4 could obscure underlying tumors and was, therefore, assumed to be clinically relevant. When the rating differed, the results were discussed among the three graders to reach a consensus.

### Statistical analysis

We analyzed the data using SPSS Statistics version 25 (IBM). The normal distribution of the continuous variables was verified using the Shapiro–Wilk test in combination with the normal *Q*–*Q* plots. When normally distributed, data were compared using the independent *t* test (two-tailed), or when not normally distributed, the Mann–Whitney *U* test (two-tailed) was used. A Chi-squared test (two-sided) was performed when appropriate. For the visual analysis, we determined whether there was a significant difference in gradings of 1–2 (low grade) and 3–4 (high grade) between the control group and the ≥ 48 h group and between the < 48 h group and ≥ 48 h group. These dichotomic data were analyzed by calculating the odds ratio (OR) in combination with the 95% confidence intervals (CI). When the number in a group was zero, we used Fisher’s exact test. For all analyses, *p* values of < 0.05 were considered to be statistically significant.

## Results

### Patient characteristics

In the total group of 126 diabetic patients, 80 patients used metformin and were considered for inclusion in this study, 32 of whom were excluded, resulting in the overall inclusion of 48 patients on metformin. Reasons for exclusion were unclear metformin dosage (*n* = 11), patients were suffering from colonic malignancies or inflammatory bowel diseases (*n* = 6), unclear metformin discontinuation period (*n* = 5), incomplete data (*n* = 4), multiple scans of which only the first scan was included (*n* = 3), scans did not include the abdomen (*n* = 2), and technical issues with the scanner (*n* = 1). Of the 48 patients using metformin, 23 reported having discontinued < 48 h and 25 had discontinued ≥ 48 h. A total of 96 patients were included in the non-diabetic control group. Between the three groups, there were no differences in gender, BMI, and injected FDG activity. Between the < 48 h group and ≥ 48 h group, there were no differences in the daily metformin dose, age, blood glucose levels, and insulin use. As expected, patients in the two metformin groups were significantly older and had a higher blood glucose level than those in the control group (Table 1).

**Table 1:** Characteristics of the study population

Characteristics	< 48 h group	≥ 48 h group	Control group	*p *value		
				< 48 h group vs. control group	≥ 48 h group vs. control group	< 48 h group vs. ≥ 48 h group
Number of patients	23	25	96	N/A	N/A	N/A
Age (years), median (25th–75th perc.)	70.0 (63.0–75.0)	69.0 (63.0–77.5)	63.0 (57.0–70.0)	0.001*	0.015*	0.796
Gender, *n* (%)	Male: 17 (73.9%) Female: 6 (26.1%)	Male: 13 (52.0%) Female: 12 (48.0%)	Male: 53 (55.2%) Female: 43 (44.8%)	0.156	0.824	0.145
BMI (kg/m^2^), median (25th–75th perc.)	27.7 (24.7–31.8)	26.6 (25.3–30.8)	26.2 (24.0–29.0)	0.214	0.212	0.919
Injected FDG activity (MBq/kg), median (25th–75th perc.)	3.6 (3.2–4.8)	4.1 (3.3–5.1)	4.0 (3.3–5.0)	0.595	0.992	0.683
Blood glucose level (mmol/L), median (25th–75th perc.)	9.0 (7.4–11.3)	8.5 (6.5–9.5)	5.3 (5.1–6.0)	0.001*	0.001*	0.238
Insulin usage, *n* (%)	Yes: 9 (39.1%) No: 14 (60.9%) Unknown: 0 (–)	Yes: 6 (24.0%) No: 15 (60.0%) Unknown: 4 (16.0%)	N/A	N/A	N/A	0.535
Daily dose metformin (mg), median (25th–75th perc.)	1000 (500–2000)	1500 (1000–2000)	N/A	N/A	N/A	0.452

*N/A* not applicable, *BMI* body mass index, *FDG* [^18^F]fluorodeoxyglucose

*Statistically significant

### FDG uptake in the colon

Uptake of FDG in the colon for the four segments varied between the three groups. Overall, high-grade uptake was rarely seen in the control group, but was more common in both metformin groups, despite the patients having discontinued their metformin use. Within the metformin groups, discontinuing for a shorter period led to higher uptake than discontinuing for a longer period. A higher FDG uptake (grades 3–4) was most frequently seen in the < 48 h group [a total of 60 segments (65.2%)], followed by the ≥ 48 h group [41 segments (41.0%)], and high uptake was least frequently seen in the control group [52 segments (13.5%)].

**Fig. 1:**
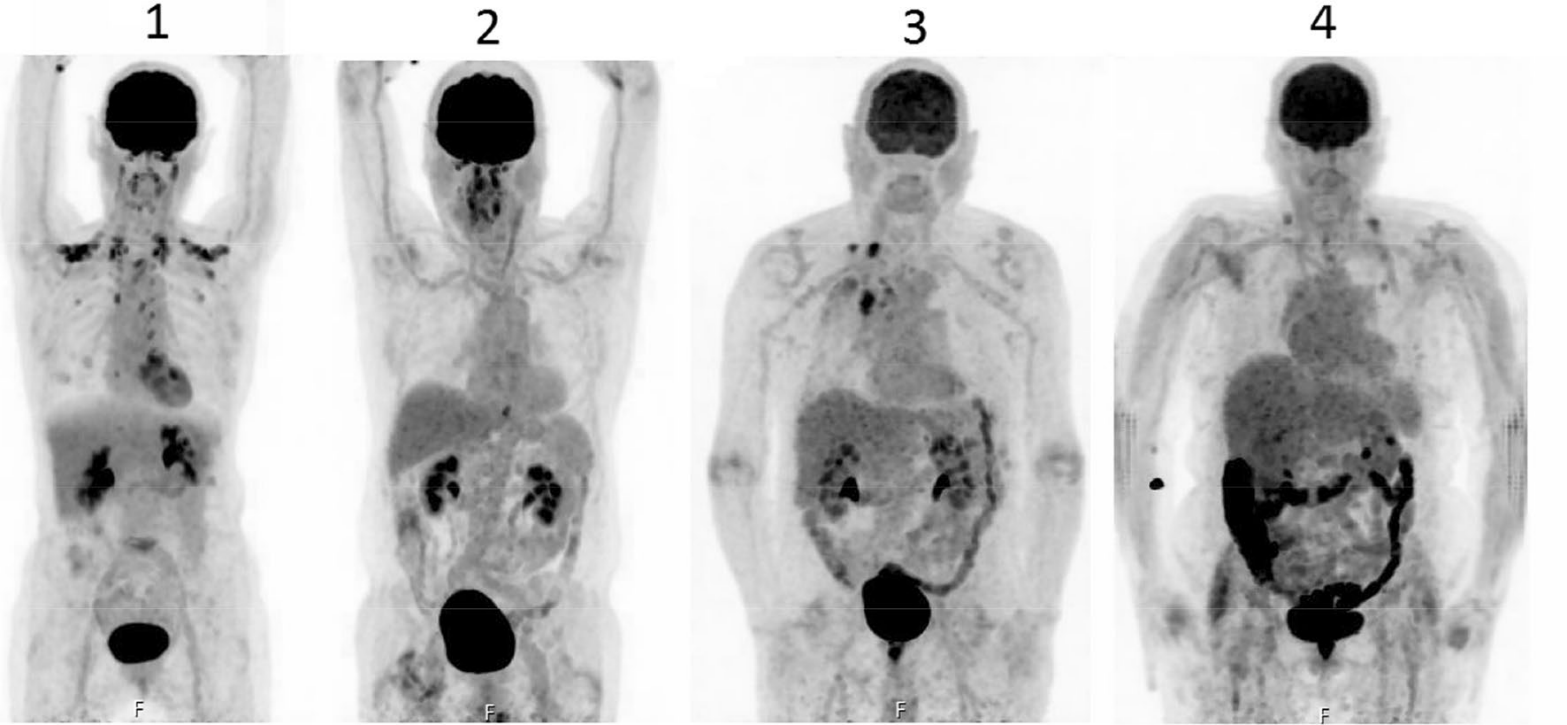
PET/CT images of four patients with different grades of FDG uptake in the colon, from left to right, corresponding to the four-point scale method of Gontier et al. [2]

The uptake of FDG also varied between the four segments (Table 2). A high uptake (grade 3–4) in each group was located in the rectosigmoid (100% in the < 48 h group, 76.0% in the ≥ 48 h group, and 21.9% in the control group). Examples of different gradings in four patients are presented in Fig. 1, while the results of the comparison of the different groups are presented below.

**Table 2:** Visual analysis of grade uptake 1–2 vs. 3–4 and OR(CI)

	Uptake grades	< 48 h group (*n* = 23)	≥ 48 h group (*n* = 25)	Control group (*n* = 96)	< 48 h group vs. control group		≥ 48 h group vs. control group		< 48 h group vs. ≥ 48 h group	
					OR (CI)	*p *value	OR (CI)	*p *value	OR(CI)	*p *value
Ascendens	1–2	15 (65.2%)	21 (84.0%)	79 (82.3%)	2.5 (0.9–6.8)	0.077	0.9 (0.3–2.9)	0.841	2.8 (0.7–11.0)	0.141
	3–4	8 (34.8%)	4 (16.0%)	17 (17.7%)						
Transversum	1–2	12 (52.2%)	21 (84.0%)	90 (93.8%)	13.8* (4.3–44.0)	0.001	2.9 (0.7–11.0)	0.128	4.8* (1.3–18.5)	0.022
	3–4	11 (47.8%)	4 (16.0%)	6 (6.3%)						
Descendens	1–2	5 (21.7%)	11 (44.0%)	88 (91.7%)	39.6* (11.6–135.1)	0.001	14.0* (4.8–40.9)	0.001	2.8 (0.8–10.0)	0.108
	3–4	18 (78.3%)	14 (56.0%)	8 (8.3%)						
Rectosigmoid	1–2	0 (–)	6 (24.0%)	75 (78.1%)	N/A	0.001**†**	11.3* (4.0–31.9)	0.001	N/A	0.023**†**
	3–4	23 (100%)	19 (76.0%)	21 (21.9%)						

*N/A* not applicable because zero in one group

*Statistically significant

^†^Statistically significant based on Fisher’s exact test

### Metformin discontinuation ≥ 48 h versus control

Our results demonstrate that even when patients discontinue metformin use for 48 h, a higher FGD uptake is still seen more frequently than in the control group. A comparison between the group in which metformin had been discontinued for ≥ 48 h and the control group revealed that the colonic FDG uptake in the ≥ 48 h group was significantly higher in the colon descendens (OR 14.0; 95% CI 4.8–40.9; *p* value: 0.001) and rectosigmoid (OR 11.3; 95% CI 4.0–31.9; *p* value: 0.001) and that uptake did not differ in the colon ascendens (OR 0.9; 95% CI 0.3–2.9; *p* value: 0.841) and transversum (OR 2.9; 95% CI 0.7–11.0; *p* value: 0.128) (Table 2).

### Metformin discontinuation ≥ 48 h versus metformin discontinuation < 48 h

Our results show that a higher uptake is seen more frequently in the < 48 h group than in the ≥ 48 h group. A comparison between the group that had discontinued metformin for ≥ 48 h and the group that had discontinued it for < 48 h revealed that the FDG uptake was significantly higher in the < 48 h group in the colon transversum (OR 4.8; 95% CI 1.3–18.5; *p* value: 0.022) and rectosigmoid (*p* value: 0.023), and there was no difference between both metformin groups in both the colon ascendens (OR 2.8; 95% CI 0.7–11.0; *p* value: 0.141) and descendens (OR 2.8; 95% CI 0.8–10.0; *p* value: 0.108) (Table 2).

## Discussion

Our results demonstrate that even when discontinuing metformin for 48 h before FDG PET scanning, colonic FGD uptake remains high when compared to patients who have not taken metformin at all. In other words, discontinuation periods of 48–72 h still do not normalize colonic FDG uptake to the level observed in patients who have not been using metformin. This increased uptake was observed to be especially high in the distal segments of the colon (descendens and rectosigmoid). In the proximal segments, a discontinuation period of 48 h normalized colonic uptake to a level that did not differ to the one observed in non-diabetic patients who had not been using metformin.

Comparing the two metformin groups, we found that patients who had discontinued metformin for at least 48 h showed high uptake less frequently than patients who had discontinued metformin for less than 48 h. However, our results are not conclusive for all segments of the colon; FDG uptake in the colon ascendens and descendens did not differ between the two groups, whereas uptake in the transversum and rectosigmoid segments remained higher after the shorter period of metformin discontinuation.

Our results are in line with the results of a study by Lee et al. [9]. Although the total number of patients in the study of Lee et al. was larger (*n* = 240), the number of patients in the 48–72 h discontinuation group of that study was relatively small (*n* = 12). In our study, performed in a European population, we included more patients discontinuing metformin for at least 48 h (*n* = 25), this contributes evidence to the theory that a metformin discontinuation period of 48 h is insufficient to prevent FDG uptake in all of the colonic segments. Our results contradict the results found in a study by Hamidizadeh et al. which showed that a discontinuation period of 48 h lowered the FDG uptake in all of the colonic segments [8]. In our study, we used a different control group in comparison with the studies of Lee et al. and Hamidizadeh et al. [8, 9]. The study of Lee et al. compared their results with a diabetic non-metformin control group and the study of Hamidizadeh et al. used a control group of patients who continued metformin. The strength of our study is that we compared our results with a non-diabetic control group that had not been using metformin. This approach enabled us to investigate whether the FDG uptake was comparable to that in a group of patients where we would not expect FDG uptake due to the metformin. Furthermore, in our study, we examined the everyday practice of nuclear medicine and were able to demonstrate the impact of the current policy to discontinue metformin use 48 h prior to scanning on the actual FDG uptake.

Due to the low number of patients with a high-grade uptake per variable, we were unable to perform logistic modeling and calculate an adjusted OR [12]. A larger sample might reveal more about potential confounders. Considering the characteristics of our study population, the patients who were using metformin were older and generally had a higher blood glucose level in comparison with the patients of the control group. However, we do not expect that these characteristics influenced our results. The previous research has demonstrated that age does not influence the FDG uptake in the intestines [13]. Although a high blood glucose level can lead to increased muscle uptake and competition with FDG for tumor uptake [14], the EANM noted—based on the previous research—that fasting hyperglycemia did not influence the clinical value of the interpretation of the scan [6]. Moreover, the above-described mechanisms do not explain an increased colonic FDG uptake in metformin patients.

Although patients were asked to withhold insulin prior to the PET/CT scan, in our study, no exact data were available on when those patients discontinued their insulin use. Although hyperinsulinemia may increase uptake in muscles, there seems to be no known association of colonic FDG uptake with insulin [3, 13, 15].

As we were interested in whether discontinuation of metformin would make the colonic FDG uptake as we would expect in patients who had not been taking this drug and similar to the uptake in non-diabetic patients, we did not include a group of patients who had continued the use of metformin, as it is already known that metformin increases FDG uptake. Including such an additional group might have revealed more about the degree of lowering the FDG uptake in patients who had discontinued metformin compared with those who had continued its use.

The mechanism behind the relationship between metformin and FDG uptake in the colon remains unclear. Proposed theories include increased lactate production—and higher glucose usage—in the intestine due to treatment with metformin [16], relocation and increase of apical glucose transporters (GLUT2) [16–19], processes related to adenosine monophosphate-activated protein kinase (AMPK) activation [20, 21], and the role of the increased intestinal microbiome [22]. A mechanism that involves a long-term effect as a result of exposure to metformin—such as the upregulation of glucose transporters—might explain why FDG uptake remains high for some segments of the colon after the discontinuation of metformin. Additional research may clarify the exact mechanism behind the relationship between metformin and FDG uptake in the colon.

The results of our study raise the question of whether patients should discontinue metformin for even longer than 48 h before FDG PET procedures. However, longer discontinuation periods may not be feasible for patients and could influence their diabetic control and health, as well as generating logistic problems in patients who require urgent PET scans. As we see a benefit of discontinuing metformin use for 48 h in the more proximal segments of the colon, we still recommend discontinuing metformin 48 h before an FDG PET/CT scan. As FDG uptake—especially in the more distal segments of the colon—cannot be completely prevented, lesions might still be obscured and significant findings may still be missed.

## Conclusion

Discontinuing metformin for 48 h in FDG PET/CT results in a high uptake in some parts of the colon when compared with non-diabetic patients who have not been using metformin. Discontinuing metformin for 48 h seems to be useful for the more proximal segments of the colon, but FDG uptake remains high in the more distal segments of the colon. Discontinuing metformin for 48 h is preferable to discontinuing for shorter periods. The exact mechanism responsible for the increased FDG uptake in the colon remains unknown.
